# Cancer Incidence and Mortality in Firefighters: A State-of-the-Art Review and Meta-Analysis

**DOI:** 10.31557/APJCP.2019.20.11.3221

**Published:** 2019

**Authors:** Elpidoforos S Soteriades, Jaeyoung Kim, Costas A Christophi, Stefanos N Kales

**Affiliations:** 1 *United Arab Emirates University, College of Medicine and Health Sciences, Institute of Public Health, Al Ain, United Arab Emirates, *; 2 *Harvard T.H. Chan School of Public Health, Department of Environmental Health, Environmental and Occupational Medicine and Epidemiology (EOME), Boston, *; 6 *Cambridge Health Alliance, Employee and Industrial Medicine, Cambridge, MA, *; 5 *The Biostatistics Center, The George Washington University, Rockville, MD, USA, *; 3 *Keimyung University, College of Medicine, Department of Preventive Medicine, Daegu, Korea, *; 4 *Cyprus University of Technology, School of Health Sciences, Cyprus International Institute for Environmental and Public Health in association with Harvard School of Public Health, Limassol, Cyprus. *

**Keywords:** Cancer incidence, mortality, firefighters, review, meta-analysis

## Abstract

**Objective:**

A systematic literature review and meta-analysis was conducted on the association between firefighting and cancer.

**Methods:**

A comprehensive literature search of databases including Medline, EMBASE, Biosis, NIOSHTIC2, Web of Science, Cancerlit, and HealthStar, for the period between 1966 to January 2007, was conducted. We also retrieved additional studies by manual searching.

**Results:**

A total of 49 studies were included in the meta-analysis. We found statistically significant associations between firefighting and cancers of bladder, brain and CNS, and colorectal cancers, consistent with several previous risk estimates. We also found statistically significant associations of firefighting with non-Hodgkin’s lymphoma, skin melanoma, prostate, and testicular cancer. For kidney, Hodgkin’s lymphoma, leukemia, lymphosarcoma and reticulosarcoma, multiple myeloma, and pancreatic cancer, we found some statistically significant but less consistent results. For all other cancers evaluated (esophageal, laryngeal, oral and pharyngeal, liver and gallbladder, lung, lymphatic and hematopoietic, non-melanoma skin cancer, stomach, and urinary cancer) we did not find any statistically significant associations.

**Conclusions:**

Although our meta-analysis showed statistically significant increased risks of either cancer incidence or mortality of certain cancers in association with firefighting, a number of important limitations of the underlying studies exist, which, precluded our ability to arrive at definitive conclusions regarding causation.

## Introduction

Firefighting has been highlighted by many previous investigations and reviews as a hazardous occupation. It has been repeatedly documented that firefighters are potentially exposed to a variety of toxic chemicals at their job including certain known carcinogens. Their working environment is unique in terms of the complexity of workplace exposures because firefighters’ exposure is varied, often high, and intermittent (Melius, 2001). For example, fires vary greatly in the nature of materials burned, their size, and the weather. The nature and concentrations of airborne particles change at the fire scene and during the stage of the fire (Golden et al., 1995). Clearly, firefighting is a physically demanding job that exposes firefighters to acute hazards as well as potentially long-term cumulative exposures. Hazardous exposures for firefighters include smoke, particulate matter, carbon monoxide (CO), oxides of nitrogen (NOX), hydrogen chloride, hydrogen cyanide, sulfur dioxide (SO_2_), sulfuric acid, acrolein, and many other organic chemicals (NIOSH, 2004). Additionally, firefighters are exposed to a number of carcinogenic agents, including benzene, benzidine, dioxins, dibenzofurans, asbestos, polycyclic aromatic hydrocarbons, 1, 3-butadiene, formaldehyde, and acrylonitrile (Melius, 2001; Golden et al., 1995; Brandt-Rauf et al., 1988; Austin et al., 2001). Furthermore, given the complexity of fire smoke, firefighters may be exposed to additional carcinogens and toxins generated at the fire scene during combustion (NIOSH, 2004).

Many of these exposures cause acute effects, but others may cause chronic health problems, such as respiratory disease, cardiovascular disease, and cancer. Cancer among firefighters has been investigated in past decades, but the extent of the occupational risk of cancer among firefighters is not yet fully understood. Several mortality studies suggested an increased risk of cancer mortality in firefighters, including leukemia, brain cancer, multiple myeloma, non-Hodgkin’s lymphoma, bladder cancer, colorectal, stomach, and prostate cancer (Melius, 2001; Golden et al., 1995; Burnett et al., 1994). However, the majority of these individual studies have several limitations. Identifying associations between firefighters’ occupational exposures and cancer risk constitutes a challenging task mainly due to the small size of such studies, the short follow-up periods, the difficulties obtaining data on occupational exposure, and the usually long latency periods (10-30 years) associated with the development of different cancers (Guidotti, 2007; Chaudhry et al., 2004). Not surprisingly, the published studies are not always consistent and our knowledge base for determining the degree of occupational risk for cancer among firefighters remains incomplete. While a substantial body of original literature has been published on the association of occupational hazards of firefighting and the risk of cancer, few meta-analyses have been conducted examining the strength and consistency of cancer risk associations among firefighters (Chaudhry et al., 2004; Howe and Lindsay, 1983; LeMasters et al., 2006). 

In the current report, we undertook a systematic review and meta-analysis of existing literature in order to qualitatively and quantitatively assess the association between the occupation of firefighting and the risk of specific pre-defined cancers. In addition to deriving an overall pooled estimate of specific cancer incidence and mortality in association with firefighting, we also examined dose-response relationships using duration of employment and time since first employment (latency). We also qualitatively evaluated the possible impact of changes in personal protective equipment on cancer risk for the periods pre- and post-1990.

## Materials and Methods

We conducted a comprehensive literature search of several electronic databases with the assistance of two librarians with expertise in electronic resources. Cancerlit and HealthStar were migrated and folded into Medline. Hence, Medline, EMBASE, and Web of Science, Biosis, and NIOSHTIC2 were selected as the target databases for our search. These were searched for published papers, letters, abstracts, and review articles on the cancer risk among firefighters. Relevant articles were identified using a combined text word and Medical Subject Headings (MeSH) or subject heading search strategy. 

Our search terms included: (1) firefighter (firefighter OR Fire-fighter OR Fire fighter OR Fireman OR Firemen OR fire fighters); (2) Fires [MeSH] OR ‘Burns, Inhalation’ [MeSH] OR smoke inhalation OR Smoke Inhalation Injury [MeSH]; (3) ‘Occupational exposure’ [MeSH] OR ‘Environmental Exposure [MeSH]: before 1988’; (4) Neoplasm [MeSH] OR Cancer*[MeSH] OR Carcinoma [MeSH]. The above were combined by {(1) AND (4)} OR {(2) AND (3)} AND (4)], which intended to capture all reports on cancers due to occupational exposure to fires or the occupation of firefighting. The original search was conducted for the period between 1960 through January 2007 and was restricted to human studies published in English. We also retrieved additional studies by manually searching the bibliographies of original research reports and review articles.

Data are presented for 24 specific cancers, namely: bladder, brain and central nervous system, colorectal, colon, rectum, esophagus, larynx, oral and pharynx, kidney, liver and gallbladder, lung, Hodgkin’s lymphoma, non-Hodgkin’s lymphoma, leukemia, lymphatic and hematopoietic, lymphosarcoma and reticulosarcoma, multiple myeloma, pancreatic, prostate, malignant melanoma, non-melanoma skin cancer, stomach, testicular, and urinary cancer. For each one of these cancer sites, the relevant studies were grouped by two main outcomes, namely cancer incidence and cancer mortality (subgroup analysis), while several studies provided information and risk estimates on both outcomes. If a study provided estimates for more than one cancer site, then the information was included in the analysis of each cancer site separately.

*Selection of studies: inclusion and exclusion criteria*

The electronic search identified 387 references. After screening all abstracts that contained firefighters and risk of cancers, 139 articles were considered potentially eligible and a detailed review was conducted. An article was excluded if it was a review article without original data, or did not report quantitative effect estimates. Duplicate publications including proceedings that preceded a peer-reviewed article were identified and each original study was used only once. Our inclusion criteria were: 1) English studies with assessment of cancer risk for firefighters; 2) Cohort studies, case-control, cross-sectional, and/or surveillance studies; 3) Study population of firefighters with a comparison group; 4) Exposure captured by firefighters as an occupation or fire as an occupational exposure; and 5) A quantitative effect estimate was provided such as standardized mortality ratio (SMR), proportional mortality ratio (PMR), relative risk (RR), standardized incidence ratio (SIR), case-control odds ratio (OR), standardized relative risk (SRR), mortality odds ratio (MOR) and standardized mortality odds ratio (SMOR). One study using standardized registration ratio (SRR) was also included (Donnan, 1996). SRR is the number of cancer registrations in an area during a time period expressed as a percentage of the number of cancer cases expected in the area if age specific rates of a standard population occurred in the area of interest. 

We excluded studies in which the study population overlapped with other articles. If there was more than one article with the same or overlapping population, preference was given to the article providing more comprehensive information. The main reasons for excluding studies were overlapping populations, relevance of occupational data, and no specific information about firefighters.

A qualitative description of each study included in the meta-analysis is summarized in Appendix A categorized by study design, and presenting geographic region, years of follow-up, characteristics of study population, reference population, exposure definition, cancer outcome, and other available confounder information. Data were extracted for each study with incidence and/or mortality outcomes, cancer sites and corresponding ICD codes, effect measures, risk estimates, and confidence intervals or p-values.

*Assessment of study quality*

Methods for defining study quality in observational studies are less clearly established. Therefore, we developed quality criteria based on a modified MOOSE guideline (Stroup et al., 2000). Each individual study was scored (-1, 0, +1) on each of the following dimensions of the study design: a) selection of cases and controls, b) comparability, c) assessment of exposure and outcome, and d) data analysis and measurement. For instance, a cohort design received a score of plus one, while a zero score was assigned to a case-control study, and a score of minus one to a surveillance study. A high proportion of missing information, or lost to follow-up, was scored as negative one on the dimension of selection of cases and controls. PMR and/or MOR were considered as non-comparable outcome measures therefore they were scored negatively with respect to the dimension of data analysis and measurement. If a study reported information about smoking or other confounding factors, then the comparability section would receive a score of positive one. 

Three reviewers were involved in the quality assessment. Two researchers with expertise in the area of occupational and environmental epidemiology reviewed each article independently. Reviewers independently assigned an overall rating to each study. Any discrepancies were discussed and resolved through consensus involving a third expert reviewer. The final quantitative score was then transformed into a qualitative category for each study. Based on the qualitative assessment score the studies were categorized as weak, adequate, or good. The classification of weak was reserved for studies where the potential for bias was thought to be high, based on methods described by the authors, or where the study could not contribute data for specific cancer sites. 

Among a total of 49 studies included in the meta-analysis (Aronson et al., 1994; Baris et al., 2001; Bates et al., 1995; Bates et al., 2000; Bates et al., 2007; Beaumont et al., 1991; Burns and Swanson, 1991; Burnett et al., 1994; Delahunt et al., 1995; Demers et al., 1992; Demers et al., 1994; Deschamps et al., 1995; Dolin and Cook-Mozaffari, 1992; Donnan, 1996; Dubrow and Wegman, 1983; Elci et al., 2003; Eliopoulos et al., 1984; Feuer and Rosenman, 1986; Figgs et al., 1995; Firth et al., 1996; Gaertner et al., 2004; Gallagher et al., 1989; Giles et al., 1993; Goldberg et al., 2001; Grimes et al., 1991; Guidotti, 1993; Hansen, 1990; Kang et al., 2008; Krishnan et al., 2003; Krstev et al., 1998; Krstev et al., 1998; Lewis et al., 1982; Ma et al., 1998; Ma et al., 2005; Ma et al., 2006; Ma et al., Mastromatteo, 1959; Milham et al., Morton and Marjanovic, 1984; Muscat and Wynder, 1995; Musk et al., 1978; Peterson and Milham, 1980; Sama et al., 1990; Stang et al., 2003; Steenland et al., 1987; Teschke et al., 1997; Tornling et al., 1994; Vena and Fiedler, 1987; Zeegers et al., 2004) 20 articles were classified as good, 11 as adequate, and 18 as weak. To examine whether there was a difference in the results based only on good studies, pooled estimates were calculated for each specific cancer using all studies, using good studies only, and finally using both good and adequate studies together. Following the data extraction and quality assessment steps, the list of studies to be included in the analysis were grouped by anatomic cancer site based on the ICD codes as provided by each study’s authors. If the ICD codes were not provided, which was the case in some studies, grouping was based on the actual description of the cancer site as indicated by the text of the article itself.

*Statistical Analyses*

Pooled estimates and confidence intervals presented were derived using STATA (version 10.0). The corresponding pooled estimates and confidence intervals in the figures were calculated with the use of the Review Manager Software (Version 4.2) developed and made available to the general public by the Cochrane Collaboration Group (Review Manager, 2002). The meta-analytic procedure of the inverse variance method was used; that is, the weight given to each study is chosen to be the inverse of the variance of the effect estimate. The confidence intervals were slightly altered using the random effects model because the weights given to each study were different, especially with respect to the smaller studies. The data were entered as logarithms of the actual values but the final results are displayed on the original scale following the appropriate transformation. The logarithm of the risk estimate was included first, together with the corresponding standard error calculated for 95% confidence intervals using the following formula:

selogrisk estimate=log⁡(upper confidence limitlower confidence limit)2(1.96)

If the confidence interval was not given but a p-value was provided, then the standard error was calculated as the ratio of the risk estimate over the z-value corresponding to the reported p-value. In cases where a study did not provide a confidence interval or a p-value for the risk estimate in question, but gave the number of the observed and the expected number of cases, the confidence interval was calculated using the Byar’s method (Rothman et al., 1979). Therefore, studies which reported a zero confidence interval (Demers et al., 1994; Ma et la., 2005) were not estimable with respect to obtaining a summary risk estimate using the Review manager software. When calculating the overall risk estimate, the between-study variation was accounted for with the use of a random-effects model, which is based on the assumption that different studies are estimating different, yet related, exposure effects (Fleiss, 1993; DerSimonian and Laird, 1986). Heterogeneity was examined using the Q statistic (Cochran’s Q: the chi-square statistic for the test of heterogeneity), I2 (measure of inconsistency), as well as the Galbraith plot for graphical exploration of outliers. The I^2^ statistic is defined as (Q-df)/Q x 100%, where Q is the χ^2^ test statistic and df denotes the corresponding degrees of freedom (Higgins and Thompson, 2002). A p-value < 0.1 was considered significant for testing for heterogeneity using the Q test.

Publication bias, or the possibility that unpublished data would contradict the results of published studies, is always a potential source of bias in meta-analyses. We checked the extent of publication bias using funnel plots (Begg and Berlin, 1988) examining them for signs of asymmetry as well as by using Egger tests (Egger et al., 1997). The funnel plots for evaluating publication bias for different cancer sites in our investigation are shown in Appendix B. Sensitivity analysis for assessing the influence of individual studies on the summary risk estimate was performed by meta-regression. Using meta-regression, we computed meta-analysis estimates by omitting one study at a time. To track trends over time, a cumulative meta-analysis was used. In cumulative meta-analyses, studies were added one at a time with the sequence determined by their year of publication, in order to examine the cumulative effect on the pooled risk estimate as each article was added. All these tests for heterogeneity, publication bias, meta-regression, and cumulative meta-analysis were applied to good and adequate studies only for each cancer site.

## Results

**Figure 1:**
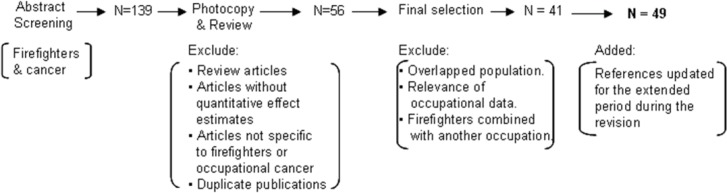
Study Selection Process

Of the 49 studies included in the meta-analysis, twenty-six were cohort studies, seventeen were case-control, and six were surveillance studies or had some other study design. Twenty-two studies were conducted in the United States, seven in Canada, four in New Zealand, two in Australia, and the remainder in European countries. Most of the studies were published after 1990; however the studies covered firefighters who worked mostly in the decades before 1990. The reference population was the corresponding general population of each study region, in most of the studies. The majority of the studies relied on death certificates for assessing cancer diagnosis, and exposure information was mostly ascertained from employment records. Recently published and newly added studies not utilized in previous meta-analytic and qualitative reviews comprised of about 20% of all studies used in our current meta-analysis. In Figure 1 we describe the study selection process that led to the final articles included in the meta-analysis. Besides the 41studies originally selected, eight studies that became available during the period of the internal/external review and met inclusion criteria were added in the revised report.

The pooled estimates and 95% confidence intervals for both cancer incidence and cancer mortality for each specific cancer examined are presented in Tables 1, 2 and 3. Forest plots for each specific cancer site are summarized in Appendix C, while in appendix D we present a comparison of our results with those of other previous qualitative and quantitative reviews. As shown in Appendix B, the vast majority of evaluation did not reveal any significant publication bias for the studies used in assessing the association for different cancer sites. An indication of publication bias was only seen for the studies related to Colon cancer, (Appendix B, page 4). There was also some marginal indication for publication bias for studies examining Rectal cancer (p-values for Begg’s and Egger tests, p = 0.10 and p = 0.07, respectively). Although tests for Leukemia, Non-Hodgkin’s lymphoma and Liver and Gallbladder cancer did not reach statistical significance, Begg’s test (p = 0.10), and Egger’s test (p = 0.09), the number of studies examined were not enough to arrive at a final conclusion with respect to publication bias. 

In Ta

ble 1 we present the results for those cancers with statistically significant findings that are consistent with previous reports examining both incidence and mortality. When all studies were examined for each specific cancer site, risk estimates for cancer of the Brain and Central Nervous System 1.26 (95% CI 1.06 – 1.50), Skin Melanoma 1.34 (95% CI 1.09 – 1.65), Colorectal cancer 1.13 (95% CI 1.06 – 1.21), and Bladder cancer 1.18 (95% CI 1.01 – 1.36), showed statistically significant associations with firefighting for both incidence and mortality (p<0.05). It is notable that the association of firefighting with Colorectal cancer was statistically significant for all three categories of studies examined (good studies only, good and adequate studies, all studies together). In addition, Non-Hodgkin’s Lymphoma, Prostate cancer and Testicular cancer were highly statistically significant. Firefighters had a 37% higher risk of developing and or dying from Non-Hodgkin’s Lymphoma 1.37 (95% CI 1.14 – 1.64) when all studies on this malignancy were pooled together (p<0.001). Similarly, firefighters had a 26% increased risk for Prostate cancer 1.26 (95% CI 1.10 – 1.45) and 68% increased risk for Testicular cancer 1.68 (95% CI 1.35 – 2.08), respectively (p<0.001). 

In addition, findings for cancers that showed some statistically significant associations with firefighting are presented in Table 2. Risk estimates for Leukemia 1.19 (95% CI 1.04 – 1.36), Multiple Myeloma 1.28 (95% CI 1.03 – 1.58), and Pancreatic cancers 1.14 (95% CI 1.01 – 1.28) showed statistically significant associations with firefighting only for the outcome of mortality and only when all studies, regardless of being good or of adequate quality, were included in the regression analyses. Ten studies were identified that addressed Hodgkin’s lymphoma incidence and/or mortality. Within a total of 10 studies, 5 studies were judged to be of good or adequate quality. The five poor quality studies used PMR as an outcome measure and were based on a small number of cases. Hodgkin’s Lymphoma showed statistically significant increased risk estimates for both mortality 1.80 (95% CI 1.27 – 2.56), and the combined outcome of incidence and mortality 1.51 (95% CI 1.13 – 2.02), only when all studies were pooled together. Similarly, Kidney cancer showed statistically significant increased risk for mortality 1.29 (95% CI 1.06 – 1.57), and for the combined outcome of incidence and mortality 1.25 (95% CI 1.02 – 1.53). In Table 3 we summarize the results for all other cancers examined (oral and pharyngeal, Laryngeal, Lung, Esophageal, Stomach, Liver and Gallbladder, Lymphatic and Hematopoietic, Urinary and Skin cancer) for which we did not find any statistically significant relationship in association with firefighting.

## Discussion

We conducted a systematic review and meta-analysis examining the association of firefighting with different cancers. Furthermore, we broadened the scope of our study by including the evaluation of dose-response relationships, duration of employment (a crude proxy of cumulative exposures), as well as a qualitative assessment of the possible impact of changes in personal protective equipment for the periods pre- and post-1990. We found an increased risk of mortality for brain and CNS cancer, and an increased risk of mortality for non-Hodgkin’s lymphoma and skin melanoma associated with firefighting. More consistent increased risks for both cancer incidence and cancer mortality among firefighters were observed for colorectal cancer as well as for colon and rectal cancer separately and for prostate and testicular cancer. Suggestive findings for an increased risk of mortality in association with firefighting were seen in leukemia, Hodgkin’s lymphoma, lymphosarcoma and reticulosarcoma, multiple myeloma, as well as for kidney and pancreatic cancers. For all other cancers examined (oral and pharyngeal, laryngeal, lung, esophageal, stomach, liver and gallbladder, lymphatic and hematopoietic, urinary and skin cancer) we did not find any statistically significant association with firefighting. In general, our statistical results are consistent with other quantitative estimates and the majority of previous reports. 

Multiple myeloma, skin melanoma and testicular cancer have been less widely studied compared to other cancers in the context of firefighting. However, some statistically significant results were found in our study and are consistent with the findings of LeMasters et al., (2006). Similar significant and consistent findings were also seen for prostate cancer and non-Hodgkin’s lymphoma. There are several reasons that some of our results may differ from those of previous reports. First, the studies included in our review compared to previous meta-analyses, were not identical. In general, because ours is the latest report, we had the opportunity to collect more underlying component studies, including recently published and newly added studies - about 20% of all studies used in our current meta-analysis- not previously examined by other reviews. We further applied finer inclusion / exclusion criteria and thus, excluded certain weaker studies used by others. In addition, the exact meta-analytic methodology was not always the same across all reports. For example; as Youakim (2006) reported in his work, he used a fixed-effects model while we used a random-effects model because we used a specific number of studies with a given heterogeneity among them. Finally, differences in the results for incidence and mortality likely relate to other various factors such as the number of studies (hence, cancer cases and power) available for each endpoint (generally more studies examined mortality than incidence) and the lethality of the cancer (e.g. most persons are expected to survive testicular cancer).

It is important to note that most of the individual component studies used in our study as well as in previous meta-analyses, examined primarily firefighters who worked prior to 1990, which severely limited our efforts to compare cancer trends before and after the introduction of improved personal protective equipment (see below). Additionally, sufficient latency periods may not have been examined in component studies of post-1990 firefighters. 

In examining the potential impact of firefighting duties, and more specifically in assessing the possible association of different workplace exposures of firefighters with the development of different tumors, one should take into account the evolving nature of firefighting duties over time and specifically during the past couple of decades. As we have been able to ascertain based on the examined literature, over the past few decades, a number of studies have examined the association between firefighting exposures and risk of cancer, in parallel with other studies reporting on the characteristics and the nature of firefighters’ workplace exposures. In order to evaluate the impact of published studies looking at the risk of cancer among firefighters in association with work-related hazards, we need to take into account a number of specific parameters. Such factors include the intensity of acute exposures associated with firefighting duties (peak exposure values), and the type and duration of different exposures as well as the frequency of exposure, in order to evaluate a cumulative measure of exposure over time. Furthermore, we need to consider changes in personal protective equipment that might have affected the current levels of exposure in comparison to those reported in previously published studies. In addition, other significant parameters that merit consideration, include the latency period between exposure and outcome; changes in firefighters’ duties; and secular generational changes. The most significant development in the recent history of firefighting has been the gradually increasing and now widespread use of the positive pressure Self Contained Breathing Apparatus (SCBA) by firefighters. This respirator has contributed to a significant decrease of smoke exposure during fire suppression operations. In addition, current significantly lower exposure levels of firefighters, especially during fire suppression operations, reflect not only on the use of improved personal protective equipment such as SCBA, but also on the decreasing number of structural fires overall, suggesting that there is not only a marked decrease in peak exposures during fires, but also a decrease in cumulative exposures as a direct effect of the decreasing number of fires. On the other hand, despite the use of SCBA during active fire suppression, Burgess et al., (2001) reported recently that firefighters who participate in overhaul operations and do not use SCBA or other respirators, exhibit acute adverse respiratory changes. Such repeated exposures may have other cumulative effects over time, potentially contributing to long-term adverse health effects including cancer. SCBA is, usually, not worn during overhaul, and although other respirators are indicated, it is not always standard practice to use them. Furthermore, we should consider that during brush and forest fires, firefighters tend to have an increased risk of pulmonary sequelae compared to those associated with structural firefighting. This discrepancy is more likely the result of decreased use / decreased level of respiratory protection in these workers than from differences in the constituents of the fire smoke to which they are exposed (Harrison et al., 1995). Nevertheless, the vast majority of studies used in the current meta-analyses, do not involve forest firefighters, and therefore, may not reflect the exposures and risks experienced by that specific subgroup of firefighting professionals.

The scope of firefighters’ duties has also been modified over time. Especially during the last two decades, firefighters are being called upon to fulfill several additional duties including the expanding nature of emergency preparedness training, hazardous materials teams, emergency medical services, as well as several other public safety duties. Such a changing work pattern for firefighters may inevitably influence the type of work-related exposures and outcomes associated with the occupation of firefighting in the future. It is also worth noting that several recent studies have documented or estimated the time spent on different duties by firefighters (Kales et al., 2003; Kales et al., 2007) and the results clearly show that fire suppression operations do not exceed, at most, 5% of the overall annual duties of firefighters (Austin et al., 2001). Similarly, the time spent on non-fire emergency duties appears to be relatively high (approximately 25%). For example, about 90% of emergency calls according to the National Fire Protection Association (NFPA) involve non-fire emergencies, primarily the provision of emergency medical services. The estimated time spent for non-emergency fire station duties is even higher (approximately 50%) in contrast to the general public notion that firefighters spend most of their time directly in fire-related duties (NFPA, 2007).

The meta-analytic data in our report, thus, examine firefighters who may have had different exposure experiences compared to the current younger generations of firefighters. Therefore, in summary, we believe that the level and frequency of fire-suppression related exposures for firefighters (peak and cumulative exposures) have gradually and significantly been reduced, over the past couple of decades, mainly due to the use of effective personal protective equipment and the reduction of fire suppression operations overall. In addition, we note that the results of the current existing literature on the association of workplace exposures with the risk of cancer in firefighters may not adequately reflect on the period marked by the consistent use of the positive pressure SCBA, which has led to the improvement of firefighters’ exposure profile. The studies available characterize primarily firefighters who worked mostly between the 1940’s to 1980’s. Thus, even though there are several risk estimates providing evidence of an increased risk of specific cancers in firefighters, and some of these results are statistically significant, we advise caution with respect to automatically adjudicating or presuming causation between firefighting and certain cancers among today’s firefighters, by directly generalizing our results based primarily on previous generations of firefighters.

Furthermore, we would like to point out additional limitations of our meta-analysis, which refer to the combination of personal risk factors for specific cancers including the family history of firefighters that, for the most part, were not controlled for in these studies. A related limitation of the present study refers to a number of relevant, secular, and generational trends observed during the past two decades, which may be associated with the risk of cancer in firefighters and should also be considered. Such parameters include the changing pattern of established cancer risk factors such as tobacco smoking, diet, and obesity among the community of firefighters and the society at large. Important changes in the above risk factors have been repeatedly documented over time within the general population, thereby inevitably affecting the status of the above factors among firefighters as well (Soteriades et al., 2002; Soteriades et al., 2005). For example, many fire departments now prohibit the hiring of firefighters who are smokers, in consideration of presumption legislation associating firefighting duties with the development of heart and lung disease being considered as occupational illnesses. Moreover, North Americans, in general, are smoking less. On the other hand, the prevalence of obesity, adversely linked with several cancers, has been gradually and steadily increasing in Western society and among firefighters, providing a mixed cancer risk factor profile. Finally, regarding secular trends, our study cannot adjust for improvements in cancer screening, accuracy of diagnosis, and treatment. As all of the above factors change and/or improve over time, it would be important in the future to focus on incidence studies of a prospective nature in order to evaluate the current levels and quality of exposures in firefighters as delineated above. Another factor worth mentioning is that we cannot account for the fact that many career firefighters hold second jobs. In some cases, these jobs are in various trades. Few, if any, studies of firefighters have collected systematic information on second jobs and other potential exposures. Thus, there is insufficient information to comment as to how, if at all, this might affect the existing body of evidence associating firefighting with the risk of different cancers.

Furthermore, several other limitations related to the available data, call for caution in making firm conclusions regarding causality. Most of the limitations are associated with the original studies used in the meta-analysis. Such weaknesses include the small number of observed cases, the limited data to assess dose-response relationships, the limited or absent information on potential confounders among firefighters (e.g. personal risk factors smoking and second jobs) with respect to the pooled estimates, and the absence of specific individual exposure data for firefighters. Finally, there are concerns regarding the different segments of the population used as reference / comparison groups in each individual study that was included in the pooled estimates of the current meta-analysis. For example, some studies compared firefighters to the general population, while others used a more limited group or even other specific occupational groups such as police officers. 

In conclusion, our results regarding a positive association of firefighting with brain and CNS cancer, bladder and colorectal cancers (colon and rectal, considered separately) are consistent with several previous summary risk estimates and/or qualitative conclusions. With regard to bladder cancer, the overall body of evidence supports a statistical increase in mortality and based on a smaller number of studies also suggests an increase in incidence. For brain and CNS cancers, a consistently suggestive pattern has been shown for increased mortality. Regarding colorectal cancer, a consistently suggestive pattern of increased risk of mortality was found along with other reviews, including colon and rectal cancers considered separately. In addition, we found statistically significant or suggestive results for non-Hodgkin’s lymphoma, skin melanoma, prostate and testicular cancer. For leukemia, Hodgkin’s lymphoma, lymphosarcoma and reticulosarcoma, multiple myeloma, pancreatic, and kidney cancer, we found some statistically significant but less consistent findings. For the remaining cancers evaluated (oral and pharyngeal, laryngeal, lung, esophageal, stomach, liver and gallbladder, lymphatic and hematopoietic, urinary and combined skin cancer), we did not find any statistical evidence of an increased risk of cancer incidence and/or mortality for firefighters.

Although our meta-analysis showed statistically significant increased risks of either cancer incidence or mortality for certain cancers in association with firefighting, a number of important limitations of the underlying studies exist, which precluded our ability to arrive at definitive conclusions regarding causation. The meta-analytic results are based primarily on observational studies from previous generations of firefighters either lacking or with only crude exposure / dose estimates. Moreover, personal risk factors (e.g. smoking) for specific cancers were not controlled in the original studies and could not be adjusted for in our meta-analysis.

**Table 1:** Pooled Estimates for the Association of Firefighting with Different Cancers

Cancer Site	Risk Estimate (95% CI) [number of studies]		
	Incidence	Mortality	Incidence and Mortality
Cancer sites with statistically significant findings and generally consistent point estimates			
Brain and CNS			
Good Studies only	0.87 (0.55 – 1.37) [4]	1.31 (0.85 – 2.01) [9]	1.19 (0.84 – 11.68) [11]
Good and Adequate	1.27 (0.89 – 1.80) [7]	1.24 (0.86 – 1.77) [11]	1.25 (0.99 – 1.59) [16]
All Studies	1.27 (0.89 – 1.80) [7]	1.26 (1.02 – 1.55)* [18]	1.26 (1.06 – 1.50)* [24]
Non-Hodgkin’s Lymphoma			
Good Studies only	1.11 (0.73 – 1.68) [3]	1.25 (0.73 – 2.61) [2]	1.20 (0.90 – 1.61) [5]
Good and Adequate	1.07 (0.91 – 1.24) [5]	1.40 (0.99 – 1.96) [3]	1.12 (0.97 – 1.28) [8]
All Studies	1.36 (0.85 – 2.18) [6]	1.44 (1.27 – 1.63) ** [7]	1.37 (1.14 – 1.64) ** [13]
Skin Melanoma			
Good Studies only	1.20 (0.86 – 1.68) [4]	0.69 (0.22 – 2.18) [2]	1.15 (0.83 – 1.59) [5]
Good and Adequate	1.10 (0.77 – 1.58) [6]	1.40 (0.46 – 4.30) [3]	1.22 (0.87 – 1.71) [8]
All Studies	1.10 (0.77 – 1.58) [6]	1.62 (1.26 – 2.10) ** [6]	1.34 (1.09 – 1.65)* [11]
Colorectal			
Good Studies only	1.04 (0.91 – 1.89) [13]	1.21 (1.07 – 1.36)* [25]	1.14 (1.03 – 1.25)* [14]
Good and Adequate	1.05 (0.93 – 1.19) [17]	1.21 (1.08 – 1.35)* [27]	1.13 (1.04 – 1.24)* [18]
All Studies	1.08 (0.95 – 1.22) [18]	1.16 (1.07 – 1.25)** [42]	1.13 (1.06 – 1.21)* [28]
Colon			
Good Studies only	1.10 (0.91 – 2.48) [4]	1.14 (0.93 – 1.39) [9]	1.11 (0.94 – 1.30) [10]
Good and Adequate	1.22 (1.05 – 1.42)* [6]	1.14 (0.93 – 1.39) [9]	1.18 (1.04 – 1.34)* [13]
All Studies	1.26 (1.10 – 1.44)**[7]	1.10 (0.99 – 1.26) [16]	1.15 (1.05 – 1.26)* [21]
Rectal			
Good Studies only	1.06 (0.80 – 1.42) [4]	1.34 (1.05 – 1.72)* [8]	1.22 (1.01 – 1.47)* [10]
Good and Adequate	0.99 (0.78 – 1.24) [5]	1.34 (1.08 – 1.67)* [9]	1.16 (0.99 – 1.36) [12]
All Studies	0.99 (0.78 – 1.24) [5]	1.25 (1.09 – 1.44) ** [16]	1.18 (1.04 – 1.34)* [19]
Bladder			
Good Studies only	1.18 (0.97 – 1.43) [6]	1.39 (0.91 – 2.11) [9]	1.24 (0.98 – 1.57) [14]
Good and Adequate	1.06 (0.88 – 1.27) [9]	1.38 (0.98 – 1.95) [11]	1.20 (0.98 – 1.45) [19]
All Studies	1.06 (0.88 – 1.27) [9]	1.28 (1.05 – 1.56)* [17]	1.18 (1.01 – 1.36)* [25]
Prostate			
Good Studies only	1.14 (0.90 – 1.45) [7]	1.20 (0.76 – 1.90) [9]	1.17 (0.90 – 1.53) [15]
Good and Adequate	1.15 (1.01 – 1.32)* [9]	1.20 (0.76 – 1.90) [9]	1.19 (0.98 – 1.44) [17]
All Studies	1.21 (1.04 – 1.42)* [11]	1.27 (1.01 – 1.61)* [16]	1.26 (1.10 – 1.45)** [26]
Testicular			
Good Studies only	1.58 (1.23 – 2.03) ** [3]	2.52 (0.67 – 9.49) [1]	1.61 (1.26 – 2.06) ** [4]
Good and Adequate	1.73 (1.31 – 2.27) ** [7]	2.52 (0.67 – 9.49) [1]	1.73 (1.35 – 2.23) ** [8]
All Studies	1.73 (1.31 – 2.27) ** [7]	1.63 (0.60 – 4.40) [3]	1.68 (1.35 – 2.08) ** [10]

Risk estimates and confidence intervals in all tables were derived using STATA; * P < 0.05; ** P < 0.001

**Table 2:** Pooled Estimates for the Association of Firefighting with Certain Cancers

	Risk Estimate (95% CI) [number of studies]		
Cancer Site	Incidence	Mortality	Incidence and Mortality
Cancer sites with some statistically significant findings			
Leukemia			
Good Studies only	0.82 (0.55 – 1.23) [2]	0.96 (0.74 – 1.26) [5]	0.92 (0.73 – 1.15) [7]
Good and Adequate	0.95 (0.70 – 1.30) [4]	0.98 (0.76 – 1.26) [6]	1.02 (0.88 – 1.19) [10]
All Studies	1.08 (0.77 – 1.52) [5]	1.19 (1.04 – 1.36)* [14]	1.14 (0.99 – 1.30) [19]
Hodgkin’s Lymphoma			
Good Studies only	0.77 (0.41 – 1.45) [2]	0.88 (0.25 – 3.02) [2]	0.79 (0.45 – 1.39) [4]
Good and Adequate	1.04 (0.57 – 1.89) [3]	0.88 (0.25 – 3.02) [2]	0.99 (0.61 – 1.61) [5]
All Studies	1.04 (0.57 – 1.89) [3]	1.80 (1.27 – 2.56)** [7]	1.51 (1.13 – 2.02)* [10]
Lymphosarcoma and Reticulosarcoma			
Good Studies only	1.01 (0.57 – 1.75) [1]	0.69 (0.22 – 2.18) [2]	1.08 (0.71 – 1.66) [3]
Good and Adequate	1.01 (0.57 – 1.75) [1]	1.21 (0.62 – 2.34) [2]	1.08 (0.71 – 1.66) [3]
All Studies	1.01 (0.57 – 1.75) [1]	1.58 (1.09 – 2.27)* [5]	1.38 (1.01 – 1.87)* [6]
Multiple Myeloma			
Good Studies only	0.70 (0.10 – 2.60) [1]	1.68 (0.90 – 3.12) [1]	1.68 (0.90 – 3.12) [1]
Good and Adequate	0.96 (0.72 – 1.28) [3]	1.68 (0.90 – 3.12) [1]	1.09 (0.75 – 1.57) [4]
All Studies	0.96 (0.72 – 1.28) [3]	1.28 (1.03 – 1.58)* [8]	1.17 (0.98 – 1.38) [10]
Pancreatic			
Good Studies only	0.89 (0.73 – 1.08) [7]	0.99 (0.79 – 1.24) [8]	0.96 (0.80 – 1.17) [12]
Good and Adequate	0.89 (0.73 – 1.08) [7]	0.99 (0.80 – 1.22) [9]	0.93 (0.81 – 1.08) [15]
All Studies	0.89 (0.73 – 1.08) [7]	1.14 (1.01 – 1.28)* [15]	1.06 (0.96 – 1.18) [21]
Kidney			
Good Studies only	0.74 (0.52 – 1.07) [3]	1.08 (0.58 – 2.02) [7]	0.95 (0.61 – 1.47) [10]
Good and Adequate	1.01 (0.80 – 1.27) [5]	1.08 (0.58 – 2.02) [7]	1.05 (0.79 – 1.38) [12]
All Studies	1.22 (0.76 – 1.95) [6]	1.29 (1.06 – 1.57)* [13]	1.25 (1.02 – 1.53)* [19]

* P < 0.05; ** P < 0.001

**Table 3:** Pooled Estimates for the Association of Firefighting with Different Cancers

	Risk Estimate (95% CI) [number of studies]		
Cancer Site	Incidence	Mortality	Incidence and Mortality
Cancer sites with no statistically significant associations			
Oral and Pharyngeal			
Good Studies only	0.80 (0.50 – 1.28) [2]	1.07 (0.70 – 1.63) [6]	0.96 (0.68 – 1.34) [8]
Good and Adequate	0.80 (0.50 – 1.28) [2]	1.08 (0.75 – 1.56) [7]	0.95 (0.70 – 1.30) [9]
All Studies	0.80 (0.50 – 1.28) [2]	1.04 (0.84 – 1.30) [11]	0.94 (0.77 – 1.15) [13]
Laryngeal			
Good Studies only	0.77 (0.50 – 1.18) [2]	0.68 (0.36 – 1.30) [4]	0.74 (0.52 – 1.06) [6]
Good and Adequate	0.77 (0.50 – 1.18) [2]	0.68 (0.36 – 1.30) [4]	0.74 (0.52 – 1.06) [6]
All Studies	1.21 (0.53 – 2.76) [3]	0.94 (0.67 – 1.31) [8]	1.02 (0.74 – 1.41) [11]
Lung			
Good Studies only	0.78 (0.52 – 1.18) [7]	0.96 (0.82 – 1.11) [9]	0.88 (0.74 – 1.05) [14]
Good and Adequate	0.85 (0.66 – 1.09) [10]	0.98 (0.85 – 1.13) [10]	0.92 (0.81 – 1.05) [18]
All Studies	0.85 (0.66 – 1.09) [10]	0.99 (0.90 – 1.09) [17]	0.95 (0.86 – 1.04) [25]
Esophageal			
Good Studies only	0.89 (0.49 – 1.62) [3]	0.90 (0.47 – 1.71) [5]	0.93 (0.60 – 1.43) [8]
Good and Adequate	1.09 (0.75 – 1.58) [5]	0.90 (0.47 – 1.71) [5]	1.02 (0.74 – 1.40) [10]
All Studies	1.09 (0.75 – 1.58) [5]	1.02 (0.80 – 1.31) [10]	1.06 (0.87 – 1.29) [15]
Stomach			
Good Studies only	0.87 (0.45 – 1.70) [5]	1.03 (0.85 – 1.24) [10]	0.99 (0.80 – 1.25) [13]
Good and Adequate	0.87 (0.60 – 1.26) [7]	1.03 (0.85 – 1.24) [10]	0.96 (0.80 – 1.16) [15]
All Studies	0.87 (0.60 – 1.26) [7]	1.05 (0.91 – 1.21) [17]	0.98 (0.84 – 1.13) [22]
Liver and Gallbladder			
Good Studies only	0.77 (0.41 – 1.45) [2]	1.10 (0.80 – 1.53) [7]	1.02 (0.77 – 1.37) [8]
Good and Adequate	0.91 (0.56 – 1.47) [3]	1.10 (0.80 – 1.53) [7]	1.04 (0.79 – 1.36) [9]
All Studies	0.91 (0.56 – 1.47) [3]	1.14 (0.94 – 1.38) [13]	1.11 (0.93 – 1.32) [15]
Lymphatic and Hematopoietic			
Good Studies only	0.32 (0.08 – 1.25) [1]	0.92 (0.67 – 1.26) [7]	0.85 (0.61 – 1.20) [7]
Good and Adequate	0.32 (0.08 – 1.25) [1]	0.84 (0.62 – 1.14) [8]	0.80 (0.59 – 1.10) [8]
All Studies	0.32 (0.08 – 1.25) [1]	1.11 (0.91 – 1.35) [13]	1.07 (0.87 – 1.32) [13]
Urinary			
Good Studies only	1.02 (0.33 – 3.12) [1]	0.60 (0.23 – 1.55) [2]	0.67 (0.34 – 1.35) [3]
Good and Adequate	1.02 (0.33 – 3.12) [1]	0.85 (0.47 – 1.56) [4]	0.86 (0.53 – 1.34) [5]
All Studies	1.02 (0.33 – 3.12) [1]	1.12 (0.59 – 2.12) [5]	1.10 (0.63 – 1.91) [6]
Skin cancer *			
Good Studies only	0.98 (0.60 – 1.59) [3]	1.08 (0.78 – 1.50) [4]	1.04 (0.82 – 1.31) [7]
Good and Adequate	0.98 (0.60 – 1.59) [3]	1.08 (0.78 – 1.50) [4]	1.04 (0.82 – 1.31) [7]
All Studies	0.98 (0.60 – 1.59) [3]	1.34 (0.98 – 1.83) [8]	1.18 (0.92 – 1.52) [11]

*, Includes Melanoma and Non-melanoma skin cancer.
